# The Onset and Progression of Myopia Slows in Chinese 15-Year-Old Adolescents Following Vocational Rather Than Academic School Pathways

**DOI:** 10.1167/iovs.65.10.42

**Published:** 2024-08-28

**Authors:** Yin Hu, Lirong Liao, Ian G. Morgan, Ling Jin, Mingguang He, Xiaohu Ding

**Affiliations:** 1 State Key Laboratory of Ophthalmology, Zhongshan Ophthalmic Center, Sun Yat-Sen University, Guangdong Provincial Key Laboratory of Ophthalmology and Visual Science, Guangdong Provincial Clinical Research Center for Ocular Diseases, Guangzhou, China; 2 Research School of Biology, College of Medicine, Biology and Environment, Australia National University, Canberra, Australia; 3 School of Optometry, The Hong Kong Polytechnic University, Kowloon, Hong Kong, China; 4 Research Centre for SHARP Vision (RCSV), The Hong Kong Polytechnic University, Kowloon, Hong Kong, China; 5 Centre for Eye and Vision Research (CEVR), Hong Kong, China

**Keywords:** myopia, education, academic senior high school (AHS), vocational senior high school (VHS)

## Abstract

**Purpose:**

The purpose of this study was to investigate the changes in spherical equivalent (SE) and axial length (AL) and cumulative incidence of myopia and high myopia in Chinese 15-year-old adolescents entering a non-academic stream of senior high school education.

**Methods:**

A total of 880 first-born twins with a baseline age range of 7 to 15 years were enrolled and followed annually until 18 years of age. Cycloplegic refractions and AL were examined. Educational exposure was divided into academic high school (AHS) and vocational high school (VHS) streams. A piecewise linear mixed-effects model was used to estimate the effect of education exposures on SE development, the slope before the age of 15 years (β_2_), and the slope change at the age of 15 years (β_3_) was compared between the 2 groups.

**Results:**

The curves of refractive development in a myopic direction changed in parallel in the AHS and VHS group before 15 years. For nonmyopic children, β_2_ was −0.19 and −0.20 diopters (D)/year (*P* = 0.270), and β_3_ was 0.16 and 0.14 D/year (*P* = 0.270), in the AHS and VHS groups, respectively. Among patients with myopia, β_2_ was −0.52 and −0.54 D/year (*P* = 0.500), and β_3_ was 0.37 and 0.32 D/year (*P* = 0.004), in the AHS and VHS groups, respectively. The trends in AL were similar. The 3-year cumulative incidence of myopia was 35.3% (AHS) versus 14.7% (VHS; *P* < 0.001), and that of high myopia was 5.7% and 3.3% (*P* = 0.129).

**Conclusions:**

Students undertaking a VHS rather than an AHS education have slower myopic shifts in refraction and less incident myopia after the age of 15 years.

Myopia is a common refractive error,^1^ that imposes significant economic burdens due to the need for correction and the loss of productivity due to uncorrected refractive errors.^2^^–^^4^ In addition, progression of myopia to high myopia leads to a number of irreversible blinding complications collectively known as pathological myopia,^5^^–^^7^ such as myopia-related macular degeneration, staphyloma, rhegmatogenous retinal detachment, retinal atrophy, and a number of other changes later in life. Myopic complications are major causes of legal blindness and low vision in people of working age.^8^ Due to its high prevalence and severe complications, myopia is becoming a most concerning public problem, including in China.^9^

In fact, myopia has reached pandemic levels in most countries in East Asia and Singapore in Southeast Asia.^10^ In mainland China, the prevalence of myopia among school-age children increases from less than 5% in the first grade of primary school to around 70% among junior high school graduates after 9 years of compulsory school education.^11^^–^^13^ Observational epidemiological evidence, backed up by Mendelian randomization^14^ and regression discontinuity analysis,^15^^–^^17^ has established that there is a causal relationship among intensive education, and limited time outdoors, and myopia. The parts of the world affected by the current epidemic of myopia have in common intensive education systems that produce among the highest outcomes in the Programme for International Student Assessment (PISA) surveys of international educational outcomes,^18^^,^^19^ and share cultural attitudes that lead to avoidance of time outdoors. In some countries where academic pressures are minimal, the prevalence of myopia among 15-year-olds is much less than 20%.^20^^,^^21^ Even in countries that achieve quite highly in the PISA surveys, although not reaching the stellar performances of China and Singapore, the prevalence of myopia is less than 20% at the end of 12 years of school, which suggests that quite high educational outcomes can be achieved without precipitating a myopia epidemic.^18^ Consistent with these causal relationships, ultra-Orthodox Jewish boys in Israel, who follow a very intensive education from an early age, develop a high prevalence of myopia and high myopia by the end of schooling, similar to that seen in both boys and girls in East Asia and Singapore. However, their sisters, who follow a much less rigorous curriculum, are much less myopic, with levels of myopia similar to those of children who receive a general education in non-religious schools.^10^ Previous studies have therefore confirmed that exposures during education accelerate the development of myopia. However, for obvious ethical reasons, there are no randomized intervention trials to explore whether stopping education will slow or stop the development and progression of myopia.

However, there is a natural experiment that can provide insight into the answer to this question. China’s education system, like many others, implements a 9-year compulsory education system. All school-age children who have turned 6 years of age by September first of a given year, must start primary school in that year, then receive 6 years of free primary school and 3 years of junior high school education. Most turn 15 years old in the final year of junior high school. After graduation from junior high school, all students sit for a competitive senior high school entrance examination. If the students reach a certain standard, they can enter academic senior high school (AHS), and spend the next 3 years on academic studies in preparation for the university entrance examination (the Gao Kao). If the students do not achieve that score, they can only enroll in vocational senior high school (VHS) and are unable to sit for the Gao Kao. These students spend the next 3 years learning technical skills in a variety of vocational areas, such as cooking, welding, etc.

The primary aim of the present study was to examine the hypothesis that if academic pressures are reduced once students are enrolled in the VHS, then we would predict that there should be less axial elongation and smaller myopic shifts than in the AHS stream, where the students are studying for the Gao Kao. To achieve this aim, we enrolled the first-born twins aged between 7 and 18 years from the Guangzhou Twin Eye Study, analyzed changes in refraction before and after 15 years of age (the normal age of graduation from junior high school), in students enrolled in both AHS and VHS.

## Methods

### Subjects

The methodology of the Guangzhou Twin Eye Study has been described in detail. Briefly, it is a population-based twin registry study that began in 2006. Twins with baseline ages from 7 to 15 years were enrolled and followed up annually up to 2018. Ethics approval was obtained from the Zhongshan Ophthalmic Center Ethics Review Board. The study was conducted in accordance with the tenets of the World Medical Association's Declaration of Helsinki. Written informed consent was obtained from their parents or legal guardians. In the current analysis, only the first-born twins aged at the last examination ≥16 years were included. Children with manifest strabismus, amblyopia, or nystagmus were excluded.

### Refraction and Ocular Biometric Measurements

Refractions and ocular biometric parameters were measured annually each summer holiday. All instruments were calibrated weekly throughout the study period. Ocular biometric parameters, including axial length (AL), was measured using noncontact partial-coherence laser interferometry (IOLMaster; Carl Zeiss Meditec, Germany). Measurements were taken five times. If the difference in any 2 measurements of AL was greater than 0.10 mm, the outlier was deleted and a repeated measurement was conducted.

After ocular biometric parameters were measured, all children underwent cycloplegia using cyclopentolate 1% solution (Alcon, Fort Worth, TX, USA). Two drops of cyclopentolate were administered 5 minutes apart and a third drop was given after a further 20 minutes. The pupil diameter and light response were measured by an ophthalmologist with a ruler and handheld light after an additional 15 minutes. Spherical refraction, cylinder refraction, and corneal curvature were measured using an autorefractor (Topcon KR8800; Japan). All parents underwent refraction measurement using the same auto-refractor without cycloplegia at the initial year of enrollment. Three measurements were taken. If the difference of any 2 readings was greater than 0.5 diopters (D), the outlier would be deleted and an additional reading was collected. The best corrected visual acuity was tested using a retroilluminated logarithm of the minimum angle of resolution chart with tumbling-E optotypes (Precision Vision, La Salle, IL, USA).

### Grading School Level

The names of the senior high schools that children attend were collected using a self-designed questionnaire. According to China's educational administration, the senior high schools were ranked into two types: VHS and AHS, the latter containing key level and general level schools. The students in the VHS are not allowed to sit for the National College Entrance Examination, so academic pressures are markedly reduced, whereas all students in the AHS will sit for the most competitive examination in China, so that academic pressures will continue to be high.

### Statistical Analysis and Definition

Refraction was evaluated by cycloplegic spherical equivalent (SE) defined as spherical power + 0.5 cylindrical power. Median and interquartile range (IQR), and mean ± standard deviation (M ± SD), were used to describe the non-normal parameters and normally distributed parameters, respectively.

In the coefficient estimation, and myopia or high myopia incidence calculation, only the right eye was chosen to represent the individual, because of the high correlation of SE between right and left eyes (*r* > 0.88 in each time visit, all *P* < 0.001). Myopia was defined as cycloplegic SE ⩽ −0.5 D, and high myopia was defined as cycloplegic SE ⩽ −6.0 D. The incidence of myopia was defined to be the proportion of participants who did not have myopia in the previous visits, who developed myopia over the next year, and the cumulative incidence was defined as the proportion of participants who did not have myopia in the previous visits, and who developed myopia over the next 3 years. Analogously, the cumulative incidence of high myopia was defined to be the proportion of participants with high myopia who did not have high myopia in the previous visits. We classified all samples into myopic or nonmyopic using an age-specific method. When myopia occurs at a certain age, the subsequent records were classified into the myopic group, whereas records before that age were classified into the nonmyopic group. Specifically, if the subject is myopic at the age of 9 years (the first time the measured value of SE appears as SE ⩽ −0.5 D), for the “8 to 9” progression of this subject is classified into the nonmyopic group, whereas for the “9 to 10” progression, he or she is classified as the myopia group.

We used two different methods to detect and test for change points during refractive development. First, we chose it based on our hypothesis, because we know 15 years of age is the dividing line for attending different high schools. We therefore used this age as the cutoff point empirically. Second, we used data-driven methods to detect change points during refractive development, using threshold regression.^22^ If this data-driven method also detects a change point at 15 years old, it will add support to our hypothesis.

At the age of 15 years, we tested the slope of refraction development before and after the change. The effect was examined by fitting the following piecewise linear mixed effects model.

$$\begin{array}{c} \begin{array}{c} SE_{i,j}\;=\;\beta_{1}+\beta_{2\;}Age_{i,j}+\beta_{3}\;\left(Age_{i,j}-15\right)+\beta_{4}\;gender_{i}\\ +b_{1i}+b_{2i}Age_{i,j}+b_{3i}\;\left(Age_{i,j}-15\right)+{ɛ}_{i,j} \end{array} \end{array}$$

where *i* refers to the *i*th child, *j* refers to the *j*th follow-up data for the children, *SE*_*i*,*j*_ refers to the SE of the *i*th child at the *j*th follow-up visit; *Age*_*i*,*j*_ refers to the age of the *i*th child at the *j*th follow-up visit; β_1_, β_2_, β_3_, and β_4_ are fixed effects; *b*_1*i*_, *b*_2*i*_ and *b*_3*i*_ are random effects; ε_*i*,*j*_ is random error. (*Age*_*i*,*j*_ − 15) is equal to 0 if *Age*_*i*,*j*_ < 15, and equal to *Age*_*i*,*j*_ – 15 if *Age*_*i*,*j*_ > 15.

According to the statistical theory of piecewise linear mixed model regression, β1, β2, and β3 have the following interpretations: β1 = intercept; β2 = the slope (D/year) of SE when age is less than 15; β3 = the change in the slope of SE at the age of 15 years, and β2 + β3 = the slope (D/year) of SE after age 15 years. The 95% confidence interval (95% CI) were reported. Bootstrap methods were used to test the difference of the coefficients (β2, β3, and β2 + β3) between VHS and AHS. Because the rates of myopic shift in refraction are significantly different in myopic and non-myopic individuals, we stratified the samples by myopic status – myopic or non-myopic. When myopia occurs at a certain age, the subsequent records were classified into the myopic group, whereas records before that age were classified into the non-myopic group.

All statistical tests were two-sided, and *P* < 0.05 was considered statistically significant. Statistical analyses were performed using a commercial statistical software package (STATA, version 16.0; Stata Corp., College Station, TX, USA).

## Results

A total of 880 first-born twins were enrolled in the current analysis, 583 (66.3%) of whom ultimately enrolled in an AHS. Study participants were followed up for a median of 7 years (IQR = 5–10 years). Mean age at the final visit was 17.6 ± 0.7 years, 404 (45.9%) were boys. No significant difference was found between the AHS group and the VHS group in gender or age of final visit, Table 1. The parents of the AHS students were more myopic than those of the VHS students (*t*-test *P* < 0.001).

**Table 1.** Characteristics of the Study Participants Attending Different Senior High School Types

	Vocational School (*N* = 297)	Academic School (*N* = 583)	*P* Value
Final examination age	17.6 ± 0.69	17.6 ± 0.71	0.855
Gender (M:F)	142:155	262:321	0.419
Father's SE	−0.70 ± 2.25	−1.21 ± 2.61	0.008
Mother's SE	−0.83 ± 2.21	−1.44 ± 2.92	0.002

**Figure 1.**
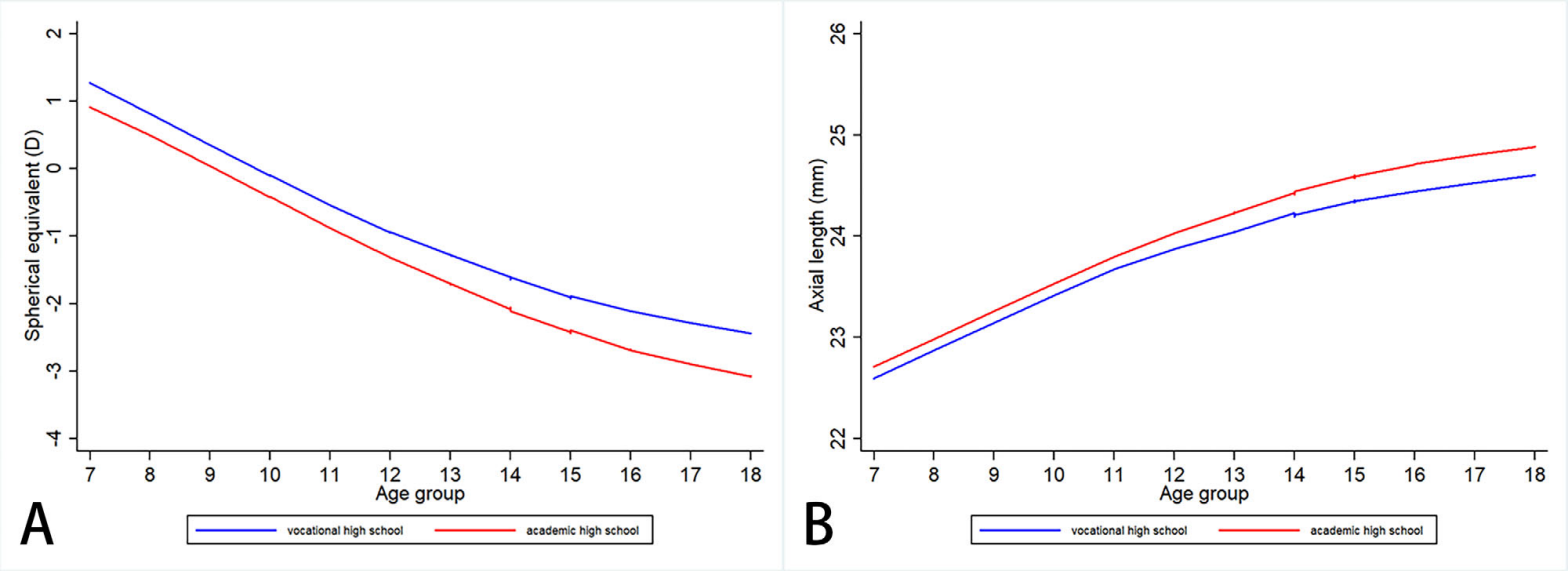
LOWESS smoothed refraction progression and axial length elongation in different senior high school streams. (**A**) Refraction and (**B**) axial length.

The changes in SE among children in the different school types are plotted in Figure 1A, using locally weighted scatterplot smoothing (LOWESS) smoothed methods. The LOWESS line suggested that refraction developed in an approximately linear manner in a myopic direction from 7 to 18 years old in the AHS group, whereas the development of myopia was more gradual in the VHS group from around the age of 12 years, and an obvious discontinuity was found in the VHS group at 15 years old. A very similar pattern of change was seen for AL (Fig. 1B).

**Figure 2.**
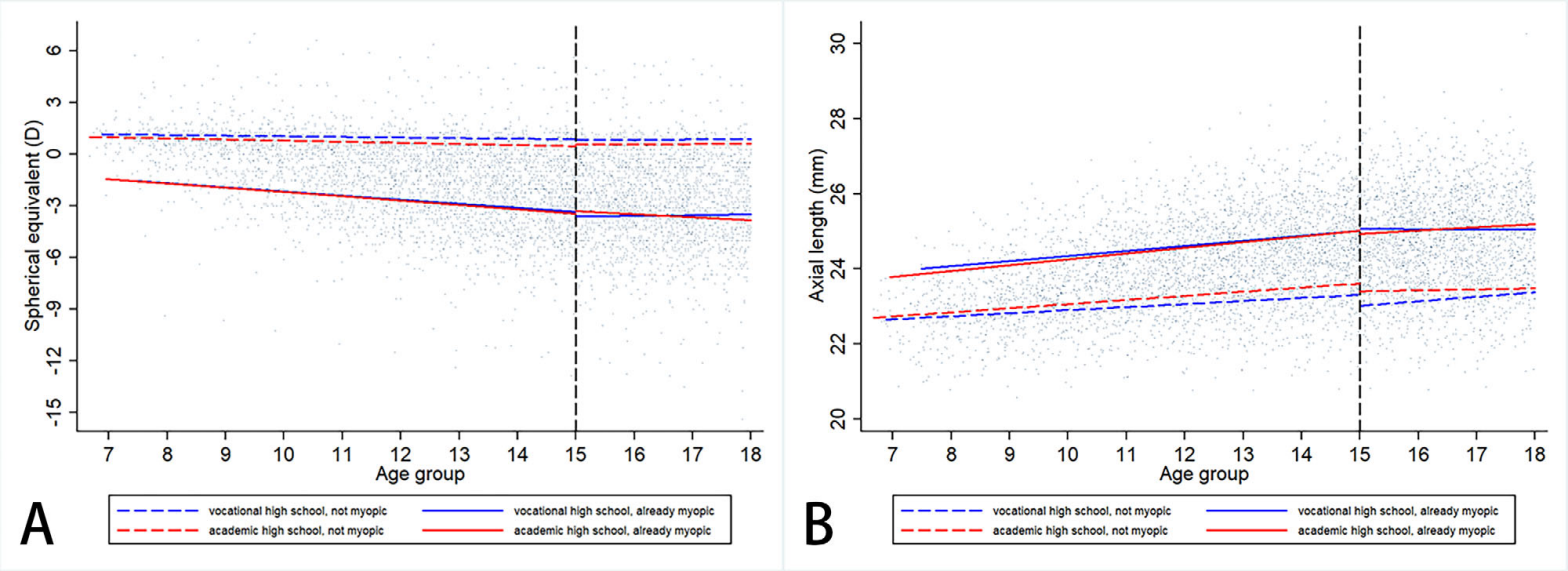
Linear regression lines of refractive progression and axial length elongation in different senior high school streams. (**A**) Refraction and (**B**) axial length. The *dotted vertical line* = at age of 15 years.

We then used a piecewise linear mixed model to quantify the slope of SE development at the point of 15 years old. In nonmyopic children, the slope of refraction before 15 years (β2) was −0.19 (95% CI = −0.21 to −0.17) and −0.20 (95% CI =−0.21 to −0.18) D/year (*P* = 0.270), the slope change at the point of 15 years (β3) was 0.16 (95% CI = 0.12 to 0.20) and 0.14 (95% CI = 0.11 to 0.18) D/year (*P* = 0.270), and was −0.03 (95% CI = −0.06 to 0.01), and −0.06 (95% CI = −0.09 to −0.03) D/year (*P* = 0.340) after 15 years old in the VHS and AHS groups, respectively. In already myopic children, the β2 was −0.52 (94% CI = −0.55 to −0.49) and −0.54 (95% CI = −0.56 to −0.52) D/year (*P* = 0.500), β3 was 0.37 (95% CI = 0.34 to 0.41) and 0.32 (95% CI = 0.29 to 0.35) D/year (*P* = 0.004), and was −0.15 (95% CI = −0.18 to −0.11), and −0.22 (95% CI = −0.24 to −0.19) D/year (*P* < 0.001) after 15 years old in the VHS and AHS groups, respectively, see Figure 2 and Table 2. For the results of AL, in nonmyopic samples, the slope of AL before 15 years (β2) was 0.15 (95% CI = 0.14 to 0.16) and 0.17 (95% CI = 0.16 to 0.18) mm/year (*P* = 0.060), the slope change at the point of 15 years (β3) was −0.12 (95% CI = −0.13 to −0.10) and −0.11 (95% CI = −0.12 to −0.10) mm/year (*P* = 0.240), and was 0.03 (95% CI = 0.02 to 0.05), and 0.06 (95% CI = 0.05 to 0.08) mm/year (*P* < 0.001) after 15 years old in the VHS and AHS groups, respectively. In already myopic samples, the β2 was 0.26 (94% CI = 0.25 to 0.28) and 0.27 (95% CI = 0.26 to 0.28) mm/year (*P* = 0.080), β3 was −0.21 (95% CI = −0.22 to −0.19) and −0.17 (95% CI = −0.18 to −0.16) mm/year (*P* < 0.001), and was 0.05 (95% CI = 0.04 to 0.07), and 0.11 (95% CI = 0.10 to 0.12) mm/year after 15 years old in the VHS and AHS groups, respectively, see Table 2. Next, we plotted the fitted line divided at the age of 15 years (see Figs. 2A, 2B). An obvious discontinuity was verified.

**Table 2.** Estimated Piecewise Linear Mixed Effects Model Relating Spherical Equivalent and Axial Length to Age for the Total Sample, Stratified by Myopia Status and High School Type

	Spherical Equivalent			Axial Length		
	*Age*_*i*,*j*_	*Age*_*i*,*j*_ − 15	–	*Age*_*i*,*j*_	*Age*_*i*,*j*_ − 15	–
	β_2_ (95% CI)	β_3_ (95% CI)	β_2_ + β_3_	β_2_ (95% CI)	β_3_ (95% CI)	β_2_ + β_3_
Nonmyopia in vocational high school	−0.19 (−0.21 to −0.17)	0.16 (0.12 to 0.20)	−0.03 (−0.06 to 0.01)	0.15 (0.14 to 0.16)	−0.12 (−0.13 to −0.10)	0.03 (0.02 to 0.05)
Nonmyopia in academic high school	−0.20 (−0.21 to −0.18)	0.14 (0.11 to 0.18)	−0.06 (−0.09 to −0.03)	0.17 (0.16 to 0.18)	−0.11 (−0.12 to −0.10)	0.06 (0.05 to 0.08)
*P* value	0.270	0.270	0.340	0.060	0.240	<0.001
Myopia in vocational high school	−0.52 (−0.55 to −0.49)	0.37 (0.34 to 0.41)	−0.15 (−0.18 to −0.11)	0.26 (0.25 to 0.28)	−0.21 (−0.22 to −0.19)	0.05 (0.04 to 0.07)
Myopia in academic high school	−0.54 (−0.56 to −0.52)	0.32 (0.29 to 0.35)	−0.22 (−0.24 to −0.19)	0.27 (0.26 to 0.28)	−0.17 (−0.18 to −0.16)	0.11 (0.10 to 0.12)
*P* value	0.500	0.004	P < 0.001	0.080	<0.001	P < 0.001

β2 = the slope (D/year) of SE when age is less than 15 years.

β3 = the change in the slope of SE at the age of 15 years.

β2 + β3 = the slope (D/year) of SE after age 15 years.

95% CI, 95% confidence interval.

To confirm the hypothesis, threshold regression was adopted to detect the change points during refraction and AL development. According to the appearance of Figure 1 seen by visual inspection, we specified two change points when building the model. The findings revealed that in the AHS stream, the 2 change points was found at 12.2 and 15.2 years old, whereas it was 12.3 and 14.2 years old in the VHS stream samples. The change point at 15.2 in the AHS and 14.2 in the VHS were aligned closely with our hypothesis.

For the change point of 12 years old, a piece-wise regression at this point was conducted, and the results are detailed in Supplementary Table S3. However, no statistically significant change in the slope of refraction development (β3) at the age of 12 years old (0.46 vs. 0.48; *P* = 0.180) was discerned between the AHS and the VHS, in the myopic sample.

**Figure 3.**
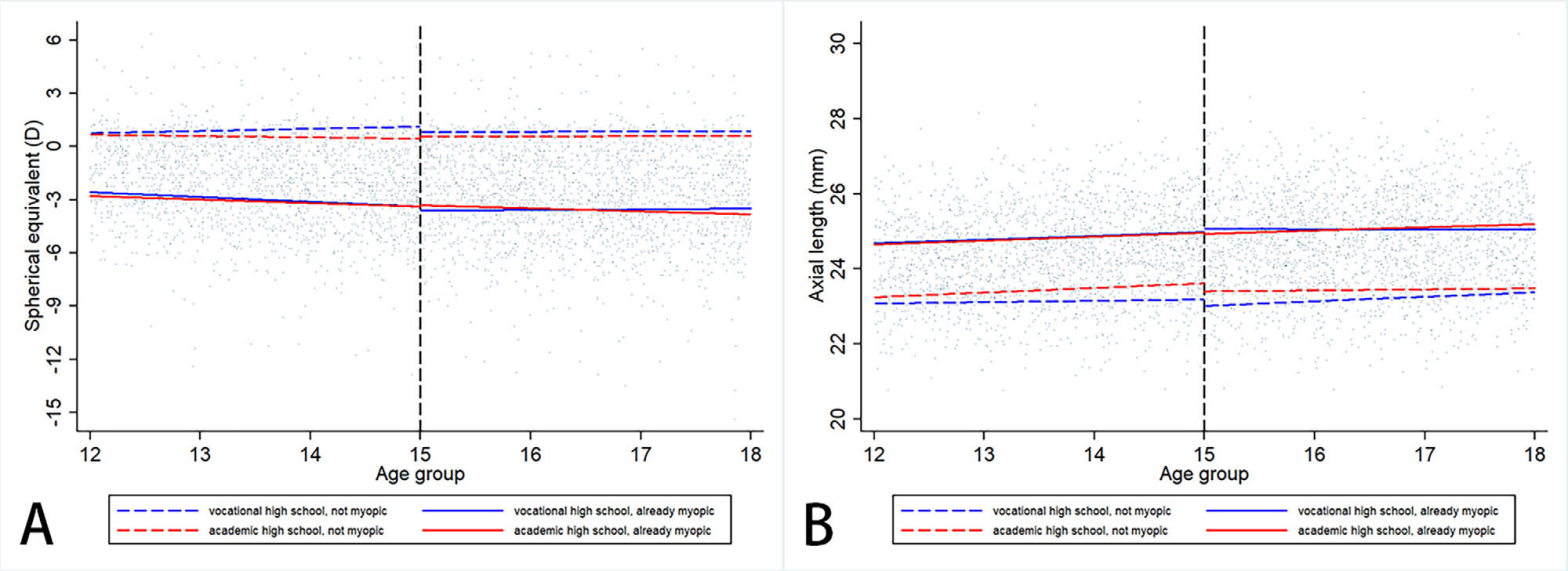
Linear regression line of refraction progression and axial length elongation in different senior high school streams, analyzed in data from 12 to 18 year old students. (**A**) Refraction and (**B**) axial length. The *dotted vertical line* = at age of 15 years.

Next, we removed samples from before the age of 12 years, and narrowed the age range of the sample to the 12 to 18-year-old group, with 15 years old still as the cutoff point. The piece-wise regression table is shown in Table 3. The linear fitted line in refraction against age by different high school type, before and after the age of 15 years, are shown in Figure 3.

**Table 3.** Estimated Piecewise Linear Mixed Effects Model Relating Spherical Equivalent and Axial Length to Age From 12 to 18 Years Old, Stratified by Myopia Status and High School Type

	Spherical Equivalent			Axial Length		
	β_2_ (95% CI)	β_3_ (95% CI)	β_2_ + β_3_	β_2_ (95% CI)	β_3_ (95% CI)	β_2_ + β_3_
Nonmyopia in vocational high school	−0.09 (−01.2 to −0.06)	0.04 (0.00 to 0.09)	−0.05 (−0.07 to −0.01)	0.08 (0.07 to 0.09)	−0.05 (−0.06 to −0.03)	0.03 (0.02 to 0.04)
Nonmyopia in academic high school	−0.13 (−0.16 to −0.11)	0.07 (0.02 to 0.12)	−0.06 (−0.09 to −0.03)	0.11 (0.10 to 0.12)	−0.05 (−0.06 to −0.03)	0.06 (0.05 to 0.07)
*P* value	0.090	0.420	0.270	<0.001	0.450	<0.001
Myopia in vocational high school	−0.44 (−0.47 to −0.40)	0.26 (0.21 to 0.30)	−0.18 (−0.21 to −0.15)	0.21 (01.9 to 0.22)	−0.13 (−0.15 −0.12)	0.08 (0.06 to 0.09)
Myopia in academic high school	−0.43 (−0.46 to −0.40)	0.17 (0.14 to 0.21)	−0.26 (−0.28 to −0.24)	0.22 (0.21 to 0.23)	−0.10 (−0.11 to −0.09)	0.12 (0.11 to 0.13)
*P* value	0.360	0.020	<0.001	0.230	<0.001	<0.001

β2 = the slope (D/year) of SE when age is less than 15 years.

β3 = the change in the slope of SE at the age of 15 years.

β2 + β3 = the slope (D/year) of SE after age 15 years.

95% CI, 95% confidence interval.

The age-specific incidence of myopia in different schools is shown in Table 4. Among those who were not myopic at the age of 15, over the next 3 years, 13 of 85 children in the VHS group and 44 of 118 children in the AHS group became myopic. If there was no refraction data at age 15 years, but data were available at age 16 years, the proportion changing from nonmyopia to myopia in the next 2 years was 1 of 8 in the VHS group and 3 of 12 in the AHS group. If refraction was not available at either 15 or 16 years, the onset of myopia after 17 years was 0 in both groups. Over the 3-year period, the cumulative incidence of myopia among students in the VHS group was 14 of 95 (14.7%), compared with 47 of 133 (35.3%) in the AHS group, with a statistically significant difference (*P* < 0.001).

**Table 4.** The Cumulative Incidence of Myopia Among Non-Myopic Students at Different Ages

	16 y	17 y	18 y	Cumulative Incidence
Academic high school				47/133 (35.3%)
15 years old (*N* = 118)	29	9	6	
16 years old (*N* = 12)^*^	—	0	3	
17 years old (*N* = 3)^#^	—	—	0	
Vocational high school				14/95 (14.7%)
15 years old (*N* = 85)	9	2	2	
16 years old (*N* = 8)^*^	—	1	0	
17 years old (*N* = 2)^#^	—	—	0	

* Did not have refraction data at 15 years old, and the visit at 16 years old was considered as baseline.

# Did not have refraction data at 15 or 16 years old, and the visit at 17 years old was considered as baseline.

Next, we analyzed the cumulative incidence of high myopia between the two groups. Among the VHS group, the sample size with SE available at the age of 15 was 261, of which 241 were not in the high myopia category. Of them, 9 progressed into high myopia at the subsequent follow-up from 15 to 18 years old. Of the 531 children who entered the AHS, 487 were not highly myopic at 15 years, and 29 developed high myopia during subsequent follow-up visits. If there were no data at age 15 years, but data were available at age 16 years, 1 of 37 children in the AHS group and 0 of 22 children in the VHS group developed high myopia. If no data were available at 15 or 16 years of age and visits were available at 17 years of age, 1 of 17 children in the AHS group and 0 of 10 children in the VHS group. The number of new cases of high myopia in different ages in different groups are listed in Table 5. Finally, over 3 years in the VHS and AHS streams, the cumulative incidence of high myopia was 31 of 541 (5.73%) in the AHS group and 9 of 273 (3.30%) in the VHS group, which showed a decrease, although the difference was not statistically significant (*P* = 0.129).

**Table 5.** The Cumulative Incidence of High Myopia Among Those Without High Myopia at Different Ages

	16 y	17 y	18 y	Cumulative Incidence
Academic high school				31/541 (5.73%)
15 years old (*N* = 487)	16	9	4	
16 years old (*N* = 37)^*^	—	1	0	
17 years old (*N* = 17)^#^	—	—	1	
Vocational high school				9/273 (3.30%)
15 years old (*N* = 241)	7	2	0	
16 years old (*N* = 22)^*^	—	0	0	
17 years old (*N* = 10)^#^	—	—	0	

* Did not have refraction data at 15 years old, and the visit at 16 years old was considered as baseline.

# Did not have refraction data at 15 or 16 years old, and the visit at 17 years old was considered as baseline.

## Discussion

For refractive development among 15 to 18 year old adolescents, compared to those who continued academic education, we found 3 important characteristics of those who discontinued academic education due to being allocated to vocational streams: (1) in non-myopic children within this group, the proportion with incident myopia was significantly lower; (2) in children with myopia, the myopic shifts in refraction and axial elongation were significantly slower; (3) in children with myopia, the proportion of children with myopia progressing to high myopia was also lower.

Because the progression of refraction and AL after myopia onset shows an age-specific pattern, so as in the slope estimation of refraction and AL, we divided each age group into myopic or non-myopic at baseline. In 2014, Sankaridurg et al.^23^ analyzed the progression rates in 508 patients with myopia in data from clinical trials, demonstrating that the annual progression shows an age-specific trend. In 6-year-old children, the annual refraction progression was about −1.0 D/year, then the rate slowed with an approximately linear trend with age. In 15-year-old children, the rate was about −0.3 D/year. No data were available after 16 years of age. Before 16 years old, our study found very similar results. After that age, the rate maintains a downward trend up to 18 years in the AHS group. However, in the VHS group, we found that entering a VHS education is associated with a reduction in myopic shifts in refraction, and reduces the incidence of myopia and high myopia. This is consistent with the evidence that children who grow up experiencing much lower educational pressures than are typical of East Asia and Singapore develop much less myopia than in this region. Conversely, in young adult Jewish boys attending Orthodox schools,^24^ the prevalence of myopia and high myopia is similar to that in young adults in East Asia, and much higher than Jewish boys attending secular schools, and much higher than in their sisters who attend religious schools, but with a less demanding academic curriculum. Our previous studies also found that an increase in grade, that is, receiving more education, rather than an increase in age is the driving force for myopia.^15^

It therefore seems likely that children who do not receive a formal education will develop little myopia – a result that is consistent with the epidemiological data available in societies of this kind.^10^ Similarly, at a later stage in the educational process, children who move out of academic into vocational pathways show little further onset and progression of myopia. The decline in progression suggests that the changed educational experience of the children in the VHS stream has led to a decline in progression rates. This has been a controversial topic, with considerable evidence suggesting that progression (that is myopic shifts in refraction in established myopes) is not regulated in the same way that myopic shifts in refraction prior to the onset of myopia are. However, there is also well-documented evidence of seasonal regulation of progression rates.^25^^,^^26^ A possible basis of the change in the students in the VHS pathway is decreased near-work and increased time outdoors, given the evidence for their role at younger ages, but this needs to be confirmed with measurement of the time spent on near-work and outdoors, preferably with objective instruments such as the Clouclip.^27^ However, depriving children of education does not provide a realistic or practical basis for preventing or controlling myopia, because considerable education is required for living in modern societies. Indeed, quality education is goal 4 of the United Nations’ Sustainable Development Goals. The challenge is therefore how to achieve quality educational outcomes for all, without generating epidemics of myopia.

From the LOWESS figures, we found that between-group differences in SE seem to begin at age 12 years, and the threshold regression results also suggest that 12 years old is the threshold. However, our piece-wise regression analysis found that the change in slope (β3) at age 12 years did not reach a statistically significant level. Interestingly, this observed difference between VHS and AHS children at the age of 12 years align well with China's education system. Chinese students typically graduate from primary school at the age of 12 years and transition to junior high school. Although junior high education is compulsory, there is still an element of school choice, with students who perform better academically often opting for better junior high schools, suggesting a higher likelihood of entering AHS in the future. We also found that the younger the onset of myopia, the greater the annual progression, which means a greater risk of having high levels of myopia as an adult, consistent with other observations that the age of onset of myopia is a major determinant of final refraction.^28^ However, attending vocational senior high school from the age of 15 years old is associated with a lower rate of annual change in refraction, eventually leading to almost zero annual change in refraction.

However, the allocation of students to the VHS or AHS streams is not random. Although it seems likely that there is a causal relationship between the postulated differences in near-work and time spent outdoors and the lesser development of myopia, given the extensive evidence for causal relationships of this kind in relation to myopia,^14^^,^^15^ our observations cannot establish causality. Children who ultimately joined the VHS steam had less severe myopia (see Fig. 1) and slower annual SE changes (see Fig. 3), over the range of 7 to 15 years of age. Different levels of parental myopia may be an important cause of this phenomenon. As can be seen from Table 1, parents of students in the AHS stream are on average more myopic. Our previous studies on this cohort have found that children with myopic parents had more severe myopia and more rapid myopia progression,^29^^,^^30^ consistent with the observations of many other studies.^31^^–^^33^ In the present study, we found that the children of more myopic parents were more likely to attend academic senior high school, and our underlying assumption in classifying students into the two streams is that the children who gain competitive admission to the academic senior high schools are more likely to have followed educational pathways that involve more educational pressure, near-work, and less time outdoors. This result is consistent with the evidence that part of the impact of parental myopia on myopia in children is mediated by parents who are more myopic and more educated, creating a more myopiagenic environment for their children. These differences prior to entering VHS mean that, whereas the results are consistent with our hypothesis, causality cannot be assumed. A randomized clinical trial in this area would be unethical, but further collection of data that shows that students in the VHS stream spend less time on near-work and more time outdoors, would certainly make it more plausible.

Only one previous study has reported on the incidence of myopia in high school students over the age of 15 years. This study,^34^ documented the incidence of myopia among Aviation Cadet Prerecruitment Class students following a selective boarding school program for entry into pilot training for the PLA Air Force. They found that the 2-year cumulative incidence was 15.9%, which is lower than our academic high school (38/118, 32.2%), but somewhat higher than our vocational high school (11/81, 12.9%). These students receive a full academic education, but in their residential program, they have a mandatory 20-minute physical training period every day, and are encouraged to participate in outdoor activities during class recesses and at other times. Previous studies have confirmed that increasing outdoor time can effectively slow the development of myopia.^35^^–^^37^ This emphasis on physical activity which frequently occurs outdoors may explain why the incidence of myopia in their study is lower than in students from academic high schools, where there is little emphasis on physical activity and time outdoors, although this may now be changing with the emphasis placed on myopia prevention in China.

Strengths of this study include that the participants are drawn from a population-based twin registry which has similar academic records and myopia prevalences to population-based sampling results.^38^ The admission rate to the academic high schools stream in our sample was about two-thirds, which is similar to the overall admission rate for Guangzhou City as a whole.^39^ Second, all samples were enrolled and examined in an era when myopia intervention, apart from eye exercises, was rarely practiced, so the refractive development was not altered by clinical intervention. However, our study has some limitations. First, there was no randomization, and thus strong conclusions about causal relationships cannot be drawn. Second, a relatively small sample size for ages less than 10 years old, which results from the design of the initial recruitment stage which covered the age range 7 to 15 years. Third, our sample is Chinese children, being educated in the Chinese education system, so our conclusion cannot be extended to the other populations, or to other education systems.

In conclusion, between 15 and 18 years old there is continued onset and progression of myopia. However, stopping academic school education coincides with significantly reduced myopic shifts in refraction in both nonmyopes and myopes, thus reducing the cumulative incidence of myopia and high myopia. It may be important to continue myopia prevention and control measures, even at this late stage in school education, particularly for students in the AHS stream, to limit further changes.

## Supplementary Material

Supplement 1
