# Supplementary material for: The Onset and Progression of Myopia Slows in Chinese 15-Year-Old Adolescents Following Vocational Rather Than Academic School Pathways
Source: Invest Ophthalmol Vis Sci. 2024 Aug 28;65(10):42. doi: 10.1167/iovs.65.10.42 (PMC11364183; doi:10.1167/iovs.65.10.42)
Supplement: Supplement 1 [file iovs-65-10-42_s001.pdf]

Supplemental information

The onset and progression of myopia slows in Chinese 15-year-old adolescents following vocational rather than academic school pathways

Yin Hu, Ian G Morgan, Ling Jin, Mingguang He, Xiaohu Ding

Supplementary table S1. The original data on annual change in spherical equivalent refraction, stratified by myopia status and high school type.

	Myopes		Non-myopes	
	Vocational (N, M±SD)	Academic (N, M±SD)	Vocational (N, M±SD)	Academic (N, M±SD)
7-8	only one record	3, -0.75±0.33	8, 0.01±0.29	37, -0.32±0.44
8-9	8, -0.80±0.49	21, -1.04±0.65	42, -0.29±0.37	92, -0.38±0.48
9-10	21, -0.80±0.38	54,-0.89±-0.46	69, -0.38±0.45	145, -0.48±0.50
10-11	43, -0.82±0.39	109, -0.84±0.40	78, -0.37±0.40	163, -0.48±0.61
11-12	66, -0.67±0.45	174, -0.72±0.51	83, -0.35±0.35	139, -0.40±0.41
12-13	102, -0.56±0.44	234, -0.57±0.47	88, -0.29±0.46	140, -0.30±0.38
13-14	129, -0.47±0.39	287, -0.51±0.42	77, -0.16±0.41	143, -0.30±0.45
14-15	154, -0.41±0.38	348, -0.37±0.38	79, -0.12±0.33	128, -0.21±0.36
15-16	173, -0.24±0.38	397, -0.32±0.44	83, -0.11±0.30	117, -0.18±0.35
16-17	179, -0.15±0.38	405, -0.25±0.34	78, 0.00±0.29	89, -0.07±0.31
17-18	159, -0.13±0.32	382, -0.24±0.35	63, -0.12±0.41	80, -0.16±0.29

Supplementary table S2. The original data of annual change in axial length, stratified by myopia status and high school type.

	myopia		Non-myopia	
	Vocational (N, M±SD)	Academic (N, M±SD)	Vocational (N, M±SD)	Academic (N, M±SD)
7-8	only one record	3, 0.43±0.20	8, 0.12±0.04	37, 0.28±0.22
8-9	8, 0.50±0.14	21, 0.55±0.24	42, 0.28±0.24	92, 0.26±0.19
9-10	21, 0.45±0.22	54, 0.44±0.18	69, 0.25±0.17	145, 0.30±0.18
10-11	43, 0.39±0.18	109, 0.42±0.13	78, 0.22±0.16	163, 0.29±0.29
11-12	66, 0.35±0.15	174, 0.34±0.24	83, 0.22±0.13	139, 0.23±0.14
12-13	102, 0.28±0.17	234, 0.29±0.15	88, 0.15±0.16	140, 0.20±0.15
13-14	129, 0.22±0.14	287, 0.25±0.12	77, 0.11±0.13	143, 0.16±0.13
14-15	154, 0.18±0.13	348, 0.18±0.12	79, 0.09±0.10	128, 0.12±0.11
15-16	173, 0.11±0.13	397, 0.15±0.12	83, 0.05±0.09	117, 0.08±0.09
16-17	179, 0.06±0.12	405, 0.13±0.12	78, 0.02±0.07	89, 0.07±0.09
17-18	159, 0.06±0.11	382, 0.10±0.10	63, 0.04±0.07	80, 0.07±0.08

Supplementary table S3. Estimated piecewise linear mixed effects model relating spherical equivalent and axial length to age, stratified by myopia status and high school type.

	Spherical equivalent			Axial length		
	$Age_{i,j}$	$Age_{i,j} - 12$	--	$Age_{i,j}$	$Age_{i,j} - 12$	--
	β_2 (95%CI)	β_2(95%CI)	$\beta_2 + \beta_3$	β_2(95%CI)	β_2(95%CI)	$\beta_2 + \beta_3$
Non-myopes in vocational high school	-0.25 (-0.28 to -0.23)	0.17 (0.14 to 0.20)	-0.08	0.20 (0.19 to 0.21)	-0.13 (-0.14 to 0.11)	0.07
Non-myopes in academic high school	-0.23 (-0.25 to -0.21)	0.10 (0.07 to 0.12)	-0.13	0.20 (0.19 to 0.21)	-0.10 (-0.11 to -0.09)	0.10
P	0.030	0.020	--	0.370	0.010	--
Myopes in vocational high school	-0.77 (-0.83 to -0.72)	0.46 (0.41 to 0.51)	-0.31	0.42 (0.40 to 0.45)	-0.28 (-0.30 to -0.26)	0.12
Myopes in academic high school	-0.83 (-0.86 to -0.79)	0.48 (0.45 to 0.52)	-0.35	0.42 (0.41 to 0.44)	-0.25 (-0.27 to -0.24)	0.17
P	0.420	0.180	--	0.160	0.020	--

β 2 = the slope (D/yr) of SE when age is less than 12 years

β3: the change in the slope of SE at the age of 12 years

β2 + β3 = the slope(D/yr) of SE after age 12 years.
